# Primary Hyperaldosteronism Presenting as Cardiac Arrest: A Case Report

**DOI:** 10.7759/cureus.86558

**Published:** 2025-06-22

**Authors:** Maryam Saleem, Maahin M Khan, Hassaan Iftikhar, Hamza Arif

**Affiliations:** 1 Nephrology, Ohio Valley Nephrology Associates, Owensboro, USA; 2 Nephrology, Washington University School of Medicine, St. Louis, USA; 3 Internal Medicine, Waterbury Hospital, Waterbury, USA; 4 Medicine, Shifa College of Medicine, Islamabad, PAK; 5 Internal Medicine, St. Francis Medical Center, Trenton, USA

**Keywords:** adrenal adenoma, cardiac arrest, hypokalemia, primary hyperaldosteronism, resistant hypertension, secondary hypertension

## Abstract

Primary hyperaldosteronism is an underdiagnosed cause of hypertension despite its increasing prevalence and strong association with cardiovascular morbidity and mortality. Early recognition is critical. Individuals with primary aldosteronism generally present with treatment-resistant hypertension and hypokalemia. Here, we present the case of a patient who experienced cardiac arrest due to severe hypokalemia and was subsequently diagnosed with primary hyperaldosteronism, confirmed through adrenal vein sampling. The condition was successfully treated with a left adrenalectomy.

## Introduction

Primary hyperaldosteronism is a frequently underdiagnosed cause of secondary hypertension. Aldosterone excess plays a central role in increasing the risk of cardiovascular complications in patients with primary aldosteronism [1]. Patients with primary aldosteronism are at heightened cardiovascular risk due to the deleterious effects of excess aldosterone, leading to structural and functional changes in the heart, which can lead to left ventricular arrhythmias and other cardiovascular events. Although patients typically present with hypertension and hypokalemia, hypokalemia is not consistently observed in clinical practice [2].

We present the case of a young male patient who was diagnosed with primary hyperaldosteronism following a cardiac arrest secondary to severe hypokalemia.

## Case presentation

A 53-year-old male patient with no known past medical history presented to the emergency department following a cardiac arrest at a local restaurant. Cardiopulmonary resuscitation (CPR) and defibrillation were performed on-site, achieving return of spontaneous circulation (ROSC).

**Figure 1:**
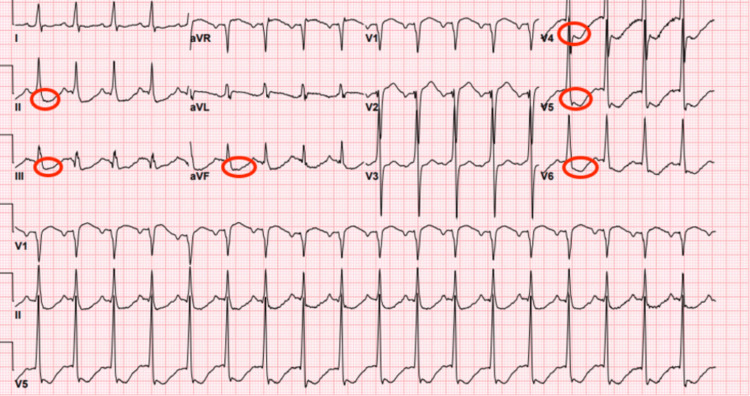
EKG with red circles indicating ST segment depression in leads II, III, aVF, V4, V5, and V6.

In the emergency department, an electrocardiogram (EKG), shown in Figure 1, revealed diffuse ST depressions. The patient was treated with aspirin, heparin, and amiodarone and underwent emergent coronary angiography, which revealed no evidence of coronary artery disease. The admission laboratory workup was notable for severe hypokalemia, with a serum potassium level of 1.7 mmol/L (reference range: 3.5-5.1 mmol/L), normal renal function, and a normal serum magnesium level.

During hospitalization, the patient developed persistent hypertension requiring intravenous antihypertensive therapy. He was initially started on a nitroglycerin infusion, which was later transitioned to clevidipine. Nephrology was consulted for the evaluation of hypertension and refractory hypokalemia. Over the course of three days, the patient required approximately 900 mEq of both oral and intravenous potassium supplementation due to persistent hypokalemia.

Given the combination of hypertension and hypokalemia, a workup for secondary causes of hypertension was initiated. The patient was discharged on nifedipine, doxazosin, spironolactone, and carvedilol, with close outpatient follow-up.

Subsequent outpatient evaluation by nephrology, endocrinology, and primary care continued. Genetic testing ruled out Liddle syndrome, apparent mineralocorticoid excess, and glucocorticoid-remediable aldosteronism. Despite being on multiple antihypertensive agents and potassium-sparing diuretics (spironolactone and irbesartan), the patient’s blood pressure remained elevated, and hypokalemia persisted. Additional medications, including irbesartan and hydralazine, were added.

**Figure 2:**
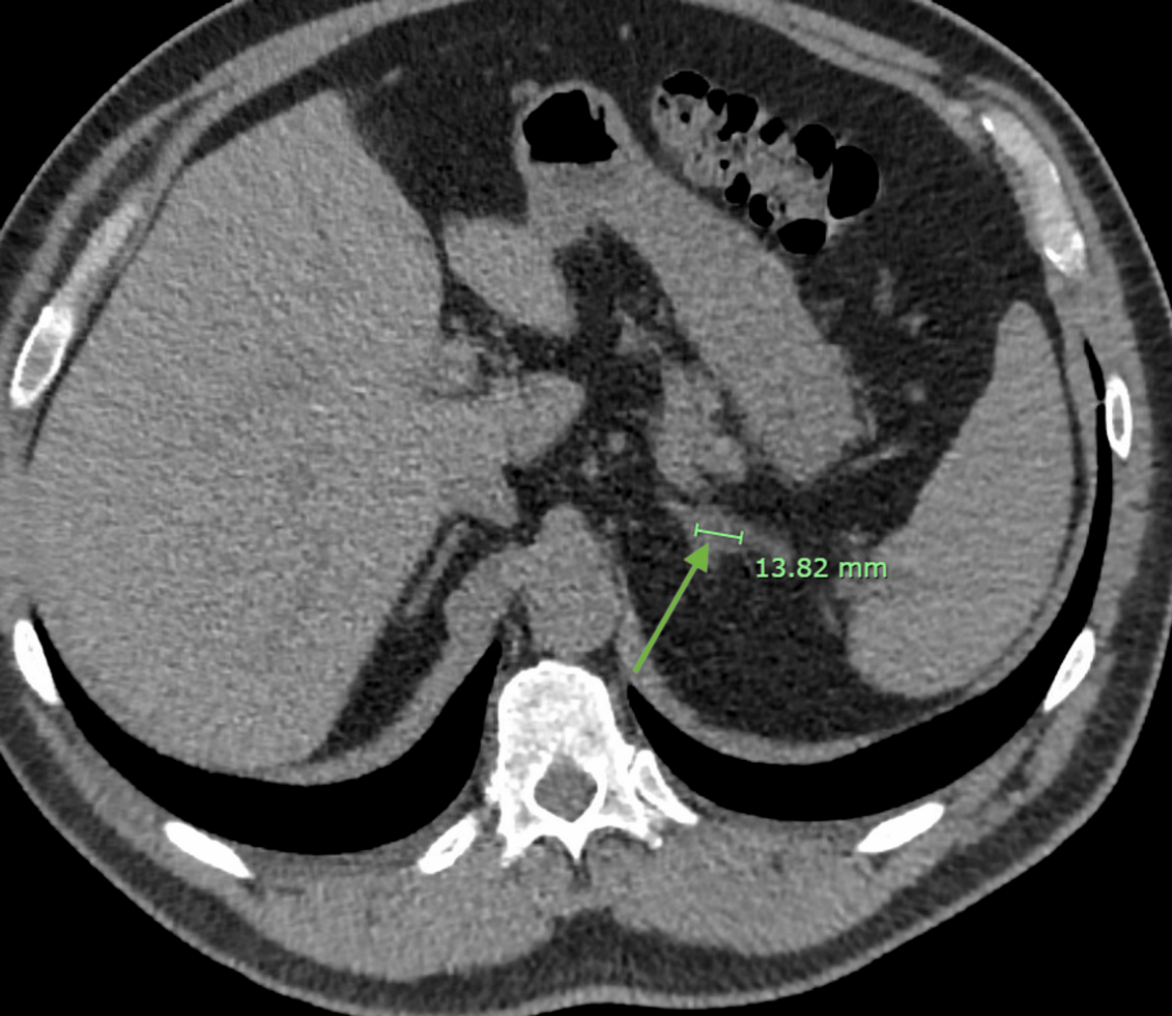
CT abdomen showing adrenal nodule (green arrow).

Further workup revealed elevated plasma aldosterone levels, suppressed plasma renin activity, and elevated 24-hour urinary aldosterone excretion, consistent with primary aldosteronism. A contrast-enhanced CT scan of the abdomen and pelvis demonstrated a left adrenal adenoma as shown in Figure 2. Adrenal vein sampling confirmed lateralization to the left adrenal gland.

The patient subsequently underwent a left adrenalectomy, resulting in the normalization of serum potassium levels and the resolution of hypertension. He was able to discontinue all antihypertensive medications postoperatively.

## Discussion

Primary hyperaldosteronism (Conn syndrome) is an underdiagnosed endocrine cause of hypertension. Individuals with primary aldosteronism generally present with treatment-resistant hypertension and hypokalemia. However, treatment-resistant hypertension is the most common initial presentation in patients with this diagnosis [1,2]. 

Primary hyperaldosteronism has been found to have poor cardiovascular, renal, and metabolic outcomes. These are mostly associated with the direct disruptive effects of hyperaldosteronism on different organ systems and relatively late diagnosis of primary aldosteronism as a cause of treatment-resistant hypertension [3,4].

Traditionally, individuals with hyperaldosteronism have been shown to experience a higher prevalence of cardiovascular complications, such as left ventricular hypertrophy, atrial fibrillation, and coronary artery disease, compared to those with essential hypertension. This association has been consistently observed in observational studies, highlighting the increased cardiovascular burden in this population. Recent cross-sectional studies further highlight these findings, reporting cardiovascular complication rates ranging between 14% and 35% in patients with hyperaldosteronism [4-8]. This highlights the need to assess the cardiovascular status of these individuals.

Symptomatic cardiovascular events in primary hyperaldosteronism are often linked to electrolyte imbalances, particularly hypokalemia. Hypokalemia is among the most common electrolyte abnormalities observed in this condition. However, it is reported in only 9-37% of patients [9,10]. EKG changes associated with hypokalemia commonly include decreased T wave amplitude, ST-segment depression, T wave inversion, prolonged PR interval, and a prolonged QTc interval. These abnormalities are frequently seen in patients with serum potassium levels ≤ 2.7 mEq/L [11].

Approximately half of individuals with primary aldosteronism who present with hypokalemia experience a delayed diagnosis. Studies have demonstrated that many of these patients have prolonged periods of hypokalemia before being diagnosed and treated for primary aldosteronism. Those with delayed diagnosis are generally older, have poorer blood pressure control, impaired renal function, and are more likely to present with cardiovascular events at the time of diagnosis [3]. Similarly, our patient initially presented with cardiac arrest despite having no significant medical history. An initial set of diagnostic tests did not reveal any underlying coronary artery disease, leading to the following event. However, further evaluation showed persistently low serum potassium and elevated blood pressure, prompting additional diagnostic workup, revealing suppressed renin and elevated aldosterone levels. A CT scan confirmed the diagnosis of primary aldosteronism, showing a left adrenal adenoma.

An evidence-based approach for patients with confirmed unilateral primary aldosteronism, such as those with an aldosterone-producing adenoma or unilateral adrenal hyperplasia, typically recommends unilateral laparoscopic adrenalectomy. This was the chosen treatment for our patient, based on his diagnosis of primary aldosteronism due to a left adrenal adenoma. Similarly, if a patient is unable or unwilling to undergo surgery, medical treatment including a mineralocorticoid receptor antagonist is recommended. Adrenalectomy effectively cures hyperaldosteronism and corrects hypokalemia in the vast majority of patients with unilateral primary aldosteronism. Also, blood pressure either normalizes or is significantly reduced in a substantial proportion of these patients [12], highlighting the impact of adrenalectomy in the management of primary aldosteronism.

Most individuals with primary hyperaldosteronism have a delayed diagnosis, which carries significant clinical implications (i.e., prolonged exposure to uncontrolled hypertension), leading to a decreased likelihood of hypertension resolution after adrenalectomy and an increased prevalence of cardiovascular complications (i.e., left ventricular hypertrophy, atrial fibrillation, and coronary artery disease) [3]. Screening for primary aldosteronism in all newly diagnosed hypertensive patients is being increasingly supported, given its potential to prevent such cardiovascular complications and enable long-term cure through early diagnosis and targeted treatment. Improving cardiovascular outcomes in individuals with primary aldosteronism is particularly important, as primary aldosteronism remains an underdiagnosed cause of hypertension. Timely recognition and management of these concerns can help prevent adverse outcomes in patients with primary aldosteronism.

## Conclusions

Patients with primary hyperaldosteronism remain at higher risk for cardiovascular complications when compared with other types of hypertension. This case highlights the importance of keeping a high index of suspicion for ruling out primary hyperaldosteronism in patients with hypokalemia and hypertension, especially the ones with refractory hypokalemia. It also signifies that successful surgical treatment of aldosterone-producing tumors can help wean patients off all antihypertensive agents. Effective management of primary hyperaldosteronism requires a multidisciplinary team-based approach to ensure accurate diagnosis, appropriate treatment selection, and optimal long-term outcomes. Collaboration among endocrinologists, radiologists, surgeons, cardiologists, nephrologists, and primary care providers enables personalized care tailored to the patient’s clinical presentation, ultimately improving blood pressure control, correcting electrolyte imbalances, and reducing cardiovascular risk.
